# Supplementary material for: Identification and analysis of individuals who deviate from their genetically-predicted phenotype
Source: PLoS Genet. 2023 Sep 21;19(9):e1010934. doi: 10.1371/journal.pgen.1010934 (PMC10564121; doi:10.1371/journal.pgen.1010934)
Supplement: S1 Text — Table A. List of 238 genes with prior evidence for a causal association with syndromes associated with stature, filtered on those with evidence of a dominant inheritance relationship. Table B. The number of individuals who are defined as deviating from their polygenic score for height using different methodologies, split by those relatively tall and relatively short for their polygenic score (total n = 158,951). Table C. Percentage overlap of individuals classified as shorter than expected for their polygenic score for height across derivation methods Table D. Percentage overlap of individuals classified as taller than expected for their polygenic score for height across derivation methods. Note, no individuals were classified as being relatively tall when using a Mahalanobis-based P-value threshold = 0.05/n. Table E. Empirical P-values for enrichment in individuals who are short relative to their genetically predicted height across all deviator definitions. Table F. Empirical P-values for enrichment in individuals who are tall relative to their genetically predicted height across all deviator definitions. No individuals were classified as being relatively tall when using a Mahalanbobis-based P-value threshold = 0.05/n. Table G. Number of individuals, and percentage of population, identified as deviating from their polygenic score for measured LDL using different methodologies. Table H. UKB Fields used to derive Q-risk measures. (PDF) [file pgen.1010934.s001.pdf]

Supplementary Information

Phenotypic criteria for filtering genes catalogued in OMIM and described as causal for syndromes associated with stature

- Short stature must not be attributable merely to failure to thrive and/or specific metabolic disturbances and/or intestinal failure/enteropathy and/or very severe disease (e.g. early lethality or severe neurologic disease

- Short stature must also be either consistent (≤ -2 SD in the great majority of patients with data recorded) or present in multiple families/sibships and accompanied by more severe short stature (-3 SD) or skeletal dysplasia (not simply poor bone quality/fractures) or brachydactyly or shortened digits (not clinodactyly or syndactyly) or disporportionate short stature or limb shortening (not simply absence of specific bones)

- Tall stature must be consistent ($> +2$ SD in the great majority of patients with data recorded) or accompanied by more severe tall stature ($> +3$ SD) or arachnodactyly

- Brachydactyly must be not just fifth finger involvement, and either consistent (present in the great majority of patients) or accompanied by consistent short stature or other skeletal dysplasias involving the spine or long bones

- Skeletal dysplasias must involve the spine or long bones (not just the skull or face) and must be accompanied by short stature or brachydactyly as a significant feature (not just clinodactyly or syndactyly), or limb or digit shortening

List of Tables

A List of 238 genes with prior evidence for a causal association with syndromes associated with stature, filtered on those with evidence of a dominant inheritance relationship. 3
B The number of individuals who are defined as deviating from their polygenic score for height using different methodologies, split by those relatively tall and relatively short for their polygenic score (total n = 158,951). 4
C Percentage overlap of individuals classified as shorter than expected for their polygenic score for height across derivation methods. 5
D Percentage overlap of individuals classified as taller than expected for their polygenic score for height across derivation methods. Note, no individuals were classified as being relatively tall when using a Mahalanobis-based P-value threshold = 0.05/n. 6
E Empirical P-values for enrichment in individuals who are short relative to their genetically predicted height across all deviator definitions. 7
F Empirical P-values for enrichment in individuals who are tall relative to their genetically predicted height across all deviator definitions. No individuals were classified as being relatively tall when using a Mahalanbobis-based P-value threshold = 0.05/n. 8
G Number of individuals, and percentage of population, identified as deviating from their polygenic score for measured LDL-C using different methodologies. 9
H UKB Data Fields used to derive Q-risk measures 10

Table A. List of 238 genes with prior evidence for a causal association with syndromes associated with stature, filtered on those with evidence of a dominant inheritance relationship.

ACAN	*CILK1*	*EZH2*	*HOXA13*	*MAGEL2*	*PDE4D*	*RPS17*	*SRCAP*
ACTB	*COL10A1*	*FAM111A*	*HOXD13*	*MAP2K1*	*PDGFRB*	*RPS19*	*SRY*
ACTG1	*COL11A1*	*FAR1*	*HRAS*	*MAP2K2*	*PHEX*	*RPS24*	*STAG2*
AFF4	*COL11A2*	*FBN1*	*HSPA9*	*MAP3K7*	*PIEZO2*	*RPS26*	*STAT5B*
AKT2	*COL1A1*	*FBN2*	*IFITM5*	*MATN3*	*PIK3R1*	*RPS28*	*TBL1XR1*
ALDH18A1	*COL1A2*	*FBXO11*	*IGF1R*	*MBD5*	*POGZ*	*RPS6KA3*	*TBX1*
ALPL	*COL2A1*	*FBXW11*	*IGF2*	*MC4R*	*POLE*	*RPS7*	*TBX3*
AMER1	*COL9A1*	*FGFR1*	*IHH*	*MECP2*	*POU1F1*	*RRAS2*	*TBX4*
ANKRD11	*COL9A2*	*FGFR2*	*IKBKG*	*MIR140*	*PPM1D*	*RUNX2*	*TBX5*
ANTXR1	*COL9A3*	*FGFR3*	*JAG1*	*MMP13*	*PPP1CB*	*SEMA3E*	*TBX6*
ARCN1	*COMP*	*FIG4*	*JAK1*	*MRAS*	*PPP2R3C*	*SGMS2*	*TCF4*
ARHGAP31	*CREBBP*	*FLNA*	*KCNJ2*	*MSX2*	*PPP3CA*	*SHOC2*	*TGFB2*
ARID1A	*CSNK2A1*	*FLNB*	*KDM3B*	*MYCN*	*PRKAR1A*	*SHOX*	*TGFB3*
ARID1B	*CYP11B1*	*FN1*	*KDM6A*	*NAA10*	*PTCH1*	*SHOX*	*TGFBR1*
ARID2	*CYP19A1*	*FZD2*	*KIF22*	*NEK1*	*PTDSS1*	*SKI*	*TGFBR2*
ATP8B1	*DDR2*	*GDF5*	*KMT2A*	*NF1*	*PTH1R*	*SLC25A24*	*THRA*
ATR	*DNMT3A*	*GH1*	*KMT2C*	*NFIX*	*PTHLH*	*SLC2A2*	*THRB*
ATRX	*DPF2*	*GHR*	*KMT2D*	*NIPBL*	*PTPN11*	*SMAD4*	*TRPS1*
BMP2	*DVL1*	*GHSR*	*KRAS*	*NLRP3*	*PUF60*	*SMARCA2*	*TRPV4*
BMPR1B	*DVL3*	*GLI2*	*LBR*	*NOG*	*QRICH1*	*SMARCA4*	*TTC21B*
BRAF	*EBF3*	*GLI3*	*LHX4*	*NOTCH1*	*RAI1*	*SMARCB1*	*USP9X*
BRCA1	*EBP*	*GMNN*	*LMBR1*	*NOTCH2*	*RBPJ*	*SMARCE1*	*WASHC5*
BRCA2	*EFNB1*	*GNAS*	*LMNA*	*NPR2*	*RIT1*	*SMC1A*	*WDR37*
BRPF1	*EP300*	*GRIN2A*	*LOX*	*NRAS*	*RNF113A*	*SMC3*	*WNT1*
BUB1B	*ERCC6*	*H1-4*	*LRP4*	*NSD1*	*ROR2*	*SNRPB*	*WNT5A*
CAMK2G	*ESR1*	*HCCS*	*LRP5*	*OFD1*	*RPL11*	*SON*	*ZBTB18*
CBL	*EVC*	*HDAC6*	*LTBP3*	*OTX2*	*RPL13*	*SOS2*	*ZC4H2*
CCNQ	*EVC2*	*HDAC8*	*LZTR1*	*P4HB*	*RPL26*	*SOST*	*ZNF148*
CDKN1C	*EXT1*	*HESX1*	*MAB21L2*	*PAX8*	*RPL35A*	*SOX11*	*SOX9*
CHD7	*EXT2*	*HMGA2*	*MAF*	*PDE3A*	*RPL5*		

Table B. The number of individuals who are defined as deviating from their polygenic score for height using different methodologies, split by those relatively tall and relatively short for their polygenic score (total n = 158,951).

Defining deviator status method	Number of individuals short for their polygenic score (% of population)	Number of individuals tall for their polygenic score (% of population)
**Mahalanobis distance** ($P < 0.001$)	150 (0.09%)	94 (0.06%)
**Mahalanobis distance** ($P < 0.05/n$)	10 (0.01%)	0 (0.00%)
**Regression residuals** (> 2 $s.d.$)	3,693 (2.32%)	3,623 (2.28%)
**Regression residuals** (> 3 $s.d.$)	415 (0.26%)	287 (0.18%)
**PS centiles** ($> Q3 + 1.5IQR$)	693 (0.44%)	523 (0.33 %)
**PS centiles** ($> Q3 + 3IQR$)	23 (0.02%)	2 (1.00×10^{-3}%)
**PS ranks** ($P < 0.001$)	340 (0.02%)	228 (0.01%)
**PS ranks** ($P < 1/10,000$)	82 (0.05%)	44 (0.01%)

s.d.=standard deviation; PS=polygenic score; IQR=interquartile range.

Table C. Percentage overlap of individuals classified as shorter than expected for their polygenic score for height across derivation methods.

	**Mahalanobis distance** ($P < 0.001$)	**Mahalanobis distance** ($P < 0.05/n$)	**Regression residuals** ($> 2s.d.$)	**Regression residuals** ($> 3s.d.$)	**PS ranks** ($P < 0.001$)	**PS ranks** ($P < 1/10000$)
**Mahalanobis distance** ($P < 0.001$)	-	-	-	-	0.37	0.47
**Mahalanobis distance** ($P < 0.05/n$)	-	-	-	-	0.03	0.11
**Regression residuals** ($> 2s.d.$)	4.06	0.27	-	-	0.09	0.02
**Regression residuals** ($> 3s.d.$)	31.40	2.40	-	-	0.78	0.20
**PS centiles** ($> Q3 + 1.5IQR$)	17.72	1.44	18.81	50.3	0.49	0.12
**PS centiles** ($> Q3 + 3IQR$)	15.32	37.54	0.62	5.54	0.21	0.99

s.d.=standard deviation; PS=polygenic score; IQR=interquartile range.

Table D. Percentage overlap of individuals classified as taller than expected for their polygenic score for height across derivation methods. Note, no individuals were classified as being relatively tall when using a Mahalanobis-based P-value threshold = 0.05/n.

	**Mahalanobis distance** ($P < 0.001$)	**Regression residuals** ($> 2s.d.$)	**Regression residuals** ($> 3s.d.$)	**PS ranks** ($P < 0.001$)	**PS ranks** ($P < 1/10000$)
**Mahalanobis distance** ($P < 0.001$)	-	-	-	0.26	0.35
**Regression residuals** ($> 2s.d.$)	2.59	-	-	0.06	0.01
**Regression residuals** ($> 3s.d.$)	21.73	-	-	0.75	0.15
**PS centiles** ($> Q3 + 1.5IQR$)	14.31	14.43	51.14	0.44	0.08
**PS centiles** ($> Q3 + 3IQR$)	2.13	0.06	0.70	0.10	0.70

s.d.=standard deviation; PS=polygenic score; IQR=interquartile range.

Table E. Empirical P-values for enrichment in individuals who are short relative to their genetically predicted height across all deviator definitions.

	**Mahalanobis distance** ($P < 0.001$)	**Mahalanobis distance** ($P < 0.05/n$)	**Regression residuals** ($> 2s.d.$)	**Regression residuals** ($> 3s.d.$)	**PS centiles** ($> Q3 + 1.5IQR$)	**PS centiles** ($> Q3{+}3IQR$)	**PS ranks** ($P < 0.001$)	**PS ranks** ($P < 1/10000$)
**LoF variant carriers**	0.01	1.00	1.00×10^{-4}	$1.00{\times}10^{-4}$	$1.00{\times}10^{-4}$	0.03	$9.00{\times}10^{-4}$	0.26
**LoF variant carriers (SS)**	$1.00{\times}10^{-4}$	1.00	$1.00{\times}10^{-4}$	$1.00{\times}10^{-4}$	$1.00{\times}10^{-4}$	$1.00{\times}10^{-4}$	$1{\times}10^{-4}$	1×10^{-4}
**Missense variant carriers**	0.16	0.75	0.38	0.33	0.27	0.62	0.49	0.94
**Missense variant carriers (SS)**	$1.84{\times}10^{-2}$	$3.43{\times}10^{-2}$	$1.00{\times}10^{-4}$	$5.00{\times}10^{-4}$	$1.00{\times}10^{-4}$	$9.58{\times}10^{-2}$	$4.45{\times}10^{-3}$	$4.45{\times}10^{-3}$
**ICD9/10 and GP**	$1.00{\times}10^{-4}$	$1.00{\times}10^{-4}$	$1.99{\times}10^{-4}$	$9.00{\times}10^{-4}$	$3.00{\times}10^{-4}$	$1.00{\times}10^{-4}$	$2.00{\times}10^{-4}$	1.00×10^{-4}
**Short (10)**	$1.00{\times}10^{-4}$	$2.94{\times}10^{-3}$	$1.00{\times}10^{-4}$	$1.00{\times}10^{-4}$	$1.00{\times}10^{-4}$	$1.00{\times}10^{-4}$	$1.00{\times}10^{-4}$	$1.00{\times}10^{-4}$
**TDI**	$1.00{\times}10^{-4}$	0.51	$1.00{\times}10^{-4}$	$1.00{\times}10^{-4}$	$1.00{\times}10^{-4}$	$3.00{\times}10^{-3}$	$1.00{\times}10^{-4}$	$1.00{\times}10^{-4}$
**SSHR**	$1.00{\times}10^{-4}$	$1.00{\times}10^{-4}$	$1.00{\times}10^{-4}$	$1.00{\times}10^{-4}$	$1.00{\times}10^{-4}$	$3.00{\times}10^{-3}$	$1.00{\times}10^{-4}$	$8.70{\times}10^{-3}$

SS=Short Stature Specific; LoF=Loss of Function; Short (10)=recalling being shorter than average at age 10; SSHR=Sitting-to-Standing Height Ratio; TDI=Townsend Deprivation Index; PS=polygenic score.

Table F. Empirical P-values for enrichment in individuals who are tall relative to their genetically predicted height across all deviator definitions. No individuals were classified as being relatively tall when using a Mahalanbobis-based P-value threshold = 0.05/n.

	Mahalanobis ($P < 0.001$)	Regression ($> 2s.d.$)	Regression ($> 3s.d.$)	PS Centiles ($> Q3 + 1.5IQR$)	PS Centiles ($> Q3{+}3IQR$)	PS Ranks ($P < 0.001$)	GRS Ranks ($P < 1/10000$)
**LoF variant carriers**	0.15	0.76	0.02	0.47	1	0.04	0.18
**LoF variant carriers (TS)**	0.02	$5.57{\times}10^{-4}$	0.07	$3.87{\times}10^{-4}$	1.00	1.00	1.00
**Missense variant carriers**	0.33	0.40	0.47	0.24	0.82	0.68	0.80
**Missense variant carriers (TS)**	1.00	0.43	0.43	0.46	0.62	0.77	1.00
**ICD9/10 and GP**	0.48	0.85	0.13	0.14	1.00	0.25	0.82
**Tall (10)**	$1.00{\times}10^{-4}$	$1.00{\times}10^{-4}$	$1.00{\times}10^{-3}$	$1.00{\times}10^{-4}$	0.38	$1.00{\times}10^{-4}$	$1.00{\times}10^{-4}$
**TDI**	0.39	0.13	0.79	0.64	1.00	0.65	0.93
**SSHR**	$1.00{\times}10^{-4}$	$1.00{\times}10^{-4}$	$1.00{\times}10^{-4}$	$1.00{\times}10^{-4}$	$1.00{\times}10^{-4}$	$1.00{\times}10^{-4}$	$2.00{\times}10^{-4}$

TS=Tall Stature Specific; TDI=Townsend Deprivation Index; SSHR=Sitting Standing Height Ratio; ICD10=International Classification of Diseases Revision 10; GP=General Practice Record Data; Tall (10)=recalling being taller than average at age 10; PS=polygenic score.

Table G. Number of individuals, and percentage of population, identified as deviating from their polygenic score for measured LDL-C using different methodologies.

Defining deviator status method	Number of Individuals with low-LDL for their polygenic score (% of population)	Number of Individuals with high-LDL for their polygenic score (% of population)
**Mahalanobis distance** ($P < 0.001$)	68 (0.05%)	90 (6.70×10^{-2}%)
**Mahalanobis distance** ($P < 0.05/n$)	1 (7.21×10^{-4}%)	2 (2.00×10^{-3}%)
**Regression residuals** (> 2 *s.d.*)	3,085 (2.28%)	3,128 (2.31%)
**Regression residuals** (> 3 *s.d.*)	287 (0.21%)	290 (0.22%)
**PS centiles** ($> Q3 + 1.5\ IQR$)	3,191 (2.36%)	3,211 (2.37%)
**PS centiles** ($> Q3 + 3\ IQR$)	13 (9.60×10^{-3}%)	15 (0.01%)
**PS ranks** ($P < 0.001$)	215 (0.16%)	222 (0.17%)
**PS ranks** ($P < 1/10,000$)	28 (0.21%)	31 (0.02%)

s.d.=standard deviation; PS=polygenic score; IQR=interquartile range.

Table H. UKB Data Fields used to derive Q-risk measures

Q-Risk Factor	UKB Data Field
**Age**	21022
**Townsend Deprivation Index**	21022
**Triglycerides**	30870
**High Density Lipoprotein (HDL)**	30760
**Systolic Blood Pressure (SBP)**	4080
**Diastolic Blood Pressure (DBP)**	4079
**Body Mass Index (BMI)**	21001
**Height**	50
**Weight**	21002
**Cigarettes per day**	2887
**Alcohol intake frequency**	1558
