# Supplementary material for: Identification and analysis of individuals who deviate from their genetically-predicted phenotype
Source: PLoS Genet. 2023 Sep 21;19(9):e1010934. doi: 10.1371/journal.pgen.1010934 (PMC10564121; doi:10.1371/journal.pgen.1010934)
Supplement: Attachment — Submitted filename: PGENETICS-D-23-00163_comments.docx [file pgen.1010934.s003.docx]

The authors identified several strategies for classifying individuals whose quantitative phenotypes were meaningfully different (misaligned) from their polygenic score predictions, and showed, using data from UK Biobank, that a small number of individuals had misaligned height and LDL-C and were enriched for other known causal factors, including rare genetic variation.

This work is significant as it highlights less commonly considered clinical uses of polygenic risk scores, including (1) identifying subsets of individuals more likely to harbor rare variants and who therefore may be good candidates for sequencing/ follow up studies and (2) using misalignment from polygenic scores as a clinical predictor of other conditions and diseases.

I have the following minor suggestions for the authors’ consideration:

1. The authors should acknowledge existing work in the binary phenotype/disease context that sought to identify similarly misclassified individuals and reached a similar conclusion about rare causal variants (PMID: 33110269). The authors’ submitted work remains meaningfully different from existing work, however, as it deals with quantitative traits.
2. The authors acknowledge that this method is currently likely to be less useful in underrepresented populations in GWAS due to limitations in predictive ability of PRS in these populations. There is currently no mention of how this method might work in a more heterogenous population, such as admixed individuals, for which PRS might be variably predictive even as predictive accuracy improves overall. Please expand on how the authors envision this method working and what care would have to be taken to use such a method to identify individuals of clinical interest in heterogenous populations in the future.
3. In Figure 1, the overplotting of points obscures the underlying distribution of the standardized polygenic scores and heights and their correlation. Please include a two dimensional density plot to better show this distribution.
4. It is not stated in the results or figure legend that individuals are removed from Figure 2B who are classified neither as aligned nor misaligned. Please provide these details so that readers can more easily understand the difference between points included in 2A and 2B.
5. A reference is missing on line 144.
