# Supplementary material for: Identification and analysis of individuals who deviate from their genetically-predicted phenotype
Source: PLoS Genet. 2023 Sep 21;19(9):e1010934. doi: 10.1371/journal.pgen.1010934 (PMC10564121; doi:10.1371/journal.pgen.1010934)
Supplement: Attachment — Submitted filename: Height_plus_LDL_Deviators_Revision_One_Comments_Final.docx [file pgen.1010934.s004.docx]

**Response to Reviewer Comments (PGENETICS-D-23-00163)**

We would first and foremost like to thank the reviewers for their kind comments on our work, and importantly for taking the time to make insightful and constructive comments on the manuscript. We have adjusted the manuscript accordingly, and commented on the changes below. Changes in the manuscript have been highlighted in yellow.

***Reviewer #1****: I love the idea of this paper. Identifying the "outliers from predicted values" and starting to characterize why they are outliers is just great and has important clinical and basic science implications (both). It is also an idea that I would expect to capture the public imagination. Overall the paper is well written and clear and the methods are solid. The choice of traits to look at is perfect. I have two concerns, however - one scientific and the other regarding the presentation.

The presentation issue is just that it its a bit disjointed. Each individual analysis is presented clearly, but there's not a narrative of what questions you are asking and why. A few more "topic sentences" at the start of each results section saying how the analysis fits into the big picture of what you're trying to understand about these people would help. Or perhaps more of an introduction to the questions you are going to ask.*

We thank the reviewer for this comment. While some sentences were already present at the beginning of some of the results sections that laid out the respective hypotheses, we have ensured all sections do the same. We have added more narrative at the start of the following sections:

***Individuals who deviate from their genetically predicted LDL-cholesterol had differing levels of common cardiovascular risk factors***

Our primary hypothesis for the LDL-C phenotype was that individuals whose LDL-C was not aligned with their polygenic prediction would have differing levels of common cardiovascular risk factors.

***Deviation from genetically predicted LDL-C increases the risk of having coronary artery disease and diabetes, even after adjusting for the effects of LDL-C, BMI and age***

Having demonstrated that individuals who deviate from their polygenic prediction had different background risk levels for cardiovascular diseases, we next hypothesised that those individuals would be more likely to be diagnosed with either coronary artery disease or diabetes

***Individuals who deviate from their genetically predicted LDL-cholesterol were more likely to be carriers of damaging exome-sequenced loss-of-function variants in*** *LDLR****,*** *APOB* ***and*** *PCSK9*

As considered for height, we finally hypothesised that individuals who were misaligned to the polygenic prediction for their phenotype would be enriched for rare genetic variants in key monogenic genes.

*My scientific issue/question is very important. Almost all of the analyses are about this group of outlier people being "enriched" for one thing or another, but there is no discussion of "enriched" compared to whom. Is it everyone else? Everyone else with matching height? Something else? For example, saying that these people are "enriched" for having been concerned about height/growth at age 10 seems confusing. Aren't these people short and tall on average compared to the whole population, so wouldn't we expect that? Or are they enriched compared to height-matched individuals who are not outliers relative to prediction? This issue is relevant to pretty much every analysis. At a minimum the comparison groups need to be explained better and the results interpreted for whether they are surprising or what they tell us. Ideally each enrichment analysis uses an appropriate comparison group so that the questions and analyses are as meaningful as possible. One additional data presentation that would be useful is a histogram of the trait value(s) for the outlier people. Are they mostly short? Mostly tall? Uniform across the whole range? (Certainly a person can be predicted short or tall and then actually end up at medium height, but it's not clear whether that would be deviant enough to land them in the outlier group.*

The fundamental purpose of our work was to use statistical approaches to classify individuals in UK Biobank as either 1) having phenotypes that were consistent with their polygenic predictor (the non-deviator group), or 2) having phenotypes that deviated away from polygenic expectation (the deviator group). Subsequently, these two groups were compared against several characteristics to determine whether there was any enrichment of a characteristic in one group of the other. The reference group (the non-deviator group) is consistent across all analyses. We apologise for not being clear in the original manuscript, and have added more text in the results section to clarify this in addition to the text present in the methods section.

We have added text clarifying the above to the beginning of the two results sections:

**Statistical analysis identifies 244 individuals misaligned to genetically predicted height**

… The remaining 109,858 individuals were classified as aligned to their polygenic prediction (residual <1SD) and formed the comparison group for enrichment analyses.

**Statistical analysis classifies 159 individuals as misaligned to their genetically predicted LDL cholesterol**

… The remaining 42,652 and 50,461 individuals were classified as aligned to their polygenic prediction (residual <1SD) and formed the comparison group for the following male and female sex-specific enrichment analyses, respectively.

We agree that the example provided of short/tall at age 10 being contrasted against adult height and polygenic deviator status is not necessarily a fair comparison. However, we do not necessarily agree that relative differences in height at age 10 are perfectly casually related to relative heights in adulthood, as earlier puberty timing could result in a relatively short adult appearing tall in adulthood. Our results at age 10 demonstrate the possibility of other factors at play (rare variants, socio-economic status etc). For the other characteristics we studied (e.g. body shape, socio-economic status, cardiovascular risk, rare coding variants), we believe the comparison is fair within the remit of the paper, setting out several hypotheses as to why an individual may be an outlier. We have added some text elucidating our thoughts to the conclusion.

We have also plotted the two figures requested (see below), but did not feel they better-presented the data than the current scatterplots.

***Reviewer #2:*** *Hawkes et al. have embarked in characterizing individuals with polygenic misalignment and investigate likely explanations using two highly polygenic traits like body height (with normal trait distribution) and cholesterol (phenotypic skewed), in individuals from the UKBIOBANK with whole-exome data and trait information (n=158,951). The authors identify 0.15% for height and 0.12% with LDL-cholesterol of individuals presenting polygenic misalignment. Further, strong associations with childhood trait presentation, disproportionate stature, diagnosis of growth syndrome, carriership of rare and LOF variants and low socioeconomic status (environmental contribution) was identified for polygenic misaligned individuals and body height. Polygenic misaligned individuals for cholesterol have associations with higher risk of cardiovascular disease and diabetes and for carriership of rare variants in LDL-C.

The study is elegantly conceived and carried out with robust methodology to address the research question. Rather than novel insight this work is a proof-of-concept demonstration useful for the interpretation of genetically-predicted traits, i.e., individuals with polygenic misalignment are more likely to carry rare genetic variation or exposed to environmental factor(s) explaining the difference between predicted and observed trait levels.

1. Can the authors discuss how the uncertainty in PRS estimates at the individual level (see* [*https://doi.org/10.1038/s41588-021-00961-5*](https://doi.org/10.1038/s41588-021-00961-5)*) affects the interpretation of their findings? Consistent classification as discordant and reproducibility across the different approach employed does not necessarily mean the "true" rank in the PRS has been determined for these individuals. This can also be an explanation why so large variation was observed according to method and threshold classification used. Applying smoothing methods and error-correction methods could provide more certainty of the PRS rank of individuals.*

Thank you for pointing this out to us – we’ve added this reference to the discussion in the ‘limitations’ section and discussed as suggested.

*2. A yet not considered explanation for the discordance can be that of undetected sample swaps or mixups… (see* [*https://doi.org/10.1093/bioinformatics/btab783*](https://doi.org/10.1093/bioinformatics/btab783)*). Likely the discordant samples of the LDL cholesterol and height efforts are not the same or the authors would have reported that. Would be could to include this connotation and succinctly justify why this is not the case here i.e., the possibility of samples swaps has been addressed.*

Thank you for pointing this tool out to us. However, we don’t think it would necessarily be the best quality-control metric for this work, as we are specifically comparing polygenic scores to phenotypes, which is also how Idefix identifies sample mix-ups. We have, however, added sample-mix-up as a potential limitation, and referenced the aforementioned paper to point out that our methods may be identifying individuals who are enriched for sample mix-up. *3. It would be informative to provide a map of the polygenic misalignment for the identified discordant individuals. Eyeballing the scatterplots, none of the individuals deviating from the polygenic expectation arise from the extremes of PRS distribution. Rather, most are located in the middle of the plot (PRS distribution), i.e., individuals have an average number of trait- increasing | decreasing alleles but then have higher or lower phenotypic values than expected for the mean of the population. When that is the case, monogenic mutations of large effects are the most likely explanation for the deviation. In absence of mutation, an influential environmental contribution would be expected (i.e., in the middle of the PRS distribution the additive polygenic contribution is averaged out resulting in heritability ~ 0). If the PRS distribution was divided into say quintiles, examine a table of clinical characteristics (like the one used in Figure 5) stratified by polygenic misalignment (yes/no). One would expect randomization of the mean value of the clinical characteristics across PRS ntiles only for the stratum without polygenic misalignment, is that observed? This analysis could help pick up influential environmental factors that are on average not randomized across ntiles in the stratum of individuals with polygenic misalignment.*

Thank you for the suggestion. It is certainly true that the majority of deviators lie in the middle of the PRS distribution – this is likely due to survivability constraints (e.g. an individual who was predicted to be tall would ‘struggle’ to be deviating-tall from their tall phenotype).

We did examine the suggestion to look at quintiles. Below, we have pasted in a table showing how the mean value of the exposures studied in our manuscript varied with quintiles of the GRS for LDL. Shown are results based on our analysis of females whose LDL-C was lower than expected).

| *EXPOSURE* | *Quintile* | *Mean in Deviators* | *Mean in Controls* | *Population Mean* | *Difference (Population mean to Deviators mean)* | *Difference (Population mean to controls mean)* |
| --- | --- | --- | --- | --- | --- | --- |
| *Age* | *Q1* | *57.86* | *56.98* | *57.00* | *-0.86* | *0.02* |
| *Age* | *Q2* | *60.67* | *56.97* | *57.00* | *-3.67* | *0.02* |
| *Age* | *Q3* | *61.58* | *57.06* | *57.00* | *-4.58* | *-0.07* |
| *Age* | *Q4* | *44.33* | *57.07* | *57.00* | *12.67* | *-0.07* |
| *Age* | *Q5* | *56.73* | *57.05* | *57.00* | *0.27* | *-0.05* |
| *Alcohol Frq* | *Q1* | *2.89* | *3.05* | *3.08* | *0.19* | *0.02* |
| *Alcohol Frq* | *Q2* | *3.00* | *3.06* | *3.08* | *0.08* | *0.02* |
| *Alcohol Frq* | *Q3* | *2.00* | *3.08* | *3.08* | *1.08* | *0.00* |
| *Alcohol Frq* | *Q4* | *3.00* | *3.09* | *3.08* | *0.08* | *-0.01* |
| *Alcohol Frq* | *Q5* | *3.50* | *3.07* | *3.08* | *-0.42* | *0.00* |
| *BMI* | *Q1* | *25.91* | *27.01* | *26.94* | *1.03* | *-0.06* |
| *BMI* | *Q2* | *25.25* | *26.97* | *26.94* | *1.69* | *-0.03* |
| *BMI* | *Q3* | *22.18* | *26.95* | *26.94* | *4.76* | *-0.01* |
| *BMI* | *Q4* | *23.82* | *26.99* | *26.94* | *3.12* | *-0.04* |
| *BMI* | *Q5* | *25.99* | *27.03* | *26.94* | *0.95* | *-0.09* |
| *Cigs per day* | *Q1* | *1.00* | *4.37* | *4.56* | *3.56* | *0.19* |
| *Cigs per day* | *Q2* | *0.00* | *4.32* | *4.56* | *4.56* | *0.23* |
| *Cigs per day* | *Q3* | *0.00* | *4.53* | *4.56* | *4.56* | *0.03* |
| *Cigs per day* | *Q4* | *0.00* | *4.43* | *4.56* | *4.56* | *0.13* |
| *Cigs per day* | *Q5* | *4.17* | *4.61* | *4.56* | *0.39* | *-0.05* |

Whilst the table does show the expected results - semi-random deviation from the population mean in the control groups, versus regular differences in the deviation group, we concluded that this way of displaying the data was less clear than how we had already presented the manuscript.  *Minor points

4. How to interpret the association in individuals with polygenic misalignment and self-reporting height at age 10 years and the disproportionate proportion? Is this an environmental contribution (i.e., growth spurt, catch-up, socioeconomic status, etc) or is it related to the effect of specific genetic factors (i.e., SHOX, NPR2, ACAN, IGF1, IGF1R, FGFR3, FBN1, EZH2 and NSD1 have also been found associated with sitting height). Similarly, this is applicable for the higher risk of cardiovascular disease and diabetes association in individuals with polygenic misalignment of LDL-C levels; is this pointing out to an environmental contribution of the influence of life-style and overall health status?*

We thank the reviewer for this comment, however we did not want to comment on causal factors too directly as many could not be empirically tested in UK Biobank, which means teasing out the genetic and non-genetic factors is more difficult. Not all deviators were carriers for pathogenic variants in the genes listed so a combination of other genetic factors and environmental factors are likely to be at play.

*6. Using the previous two points as example, authors can discuss if there is a role for gene-environment interactions in the polygenic misalignment.*

We have acknowledged the possibility of GxE interactions being a factor in driving polygenic misalignment in the discussion.

*7. Given that the likelihood of polygenic misalignment seems to be much less in the extremes of the PRS distribution, the authors can suggest from their work this can be an advantage for risk classification purposes i.e., the comparison of PRS extremes is better suited to evaluate unconfounded contrasts between phenotypes (e.g., Recall by Genotype). Also, support for the use of MR approaches.*

Our analyses do not provide evidence that using individuals in the extremes of the PRS distribution for follow-up studies is invalid.

*8. Line 352 pg 14 “This included self-reporting of bring shorter…” => being shorter*

Thank you for highlighting this – we have corrected this error. *9. Supp Table 5 misses in the footer spelling out the TDI abbreviation.*

Thank you for highlighting this – we have corrected this error.

***Reviewer #3:*** *The authors identified several strategies for classifying individuals whose quantitative phenotypes were meaningfully different (misaligned) from their polygenic score predictions, and showed, using data from UK Biobank, that a small number of individuals had misaligned height and LDL-C and were enriched for other known causal factors, including rare genetic variation.*

*This work is significant as it highlights less commonly considered clinical uses of polygenic risk scores, including (1) identifying subsets of individuals more likely to harbor rare variants and who therefore may be good candidates for sequencing/ follow up studies and (2) using misalignment from polygenic scores as a clinical predictor of other conditions and diseases.*

*I have the following minor suggestions for the authors’ consideration:*

1. *The authors should acknowledge existing work in the binary phenotype/disease context that sought to identify similarly misclassified individuals and reached a similar conclusion about rare causal variants (PMID: 33110269). The authors’ submitted work remains meaningfully different from existing work, however, as it deals with quantitative traits.*

We have added in a sentence referencing this paper as another example of rare variants causing a polygenic-disease mismatch.

1. *The authors acknowledge that this method is currently likely to be less useful in underrepresented populations in GWAS due to limitations in predictive ability of PRS in these populations. There is currently no mention of how this method might work in a more heterogenous population, such as admixed individuals, for which PRS might be variably predictive even as predictive accuracy improves overall. Please expand on how the authors envision this method working and what care would have to be taken to use such a method to identify individuals of clinical interest in heterogenous populations in the future.*

We would suggest, for admixed/genetically heterogenous populations, that deviator status/degree be calculated within groups of genetic similarity, before being recombined for enrichment analyses – this has been added to the discussion.

1. *In Figure 1, the overplotting of points obscures the underlying distribution of the standardized polygenic scores and heights and their correlation. Please include a two dimensional density plot to better show this distribution.*

Thank you for this suggestion – Figure 1 has been adjusted as requested

1. *It is not stated in the results or figure legend that individuals are removed from Figure 2B who are classified neither as aligned nor misaligned. Please provide these details so that readers can more easily understand the difference between points included in 2A and 2B.*

Apologies for the confusion – this has been resolved

1. *A reference is missing on line 144.*

The reference has been added – thanks for spotting this
