# Supplementary material for: Identification and analysis of individuals who deviate from their genetically-predicted phenotype
Source: PLoS Genet. 2023 Sep 21;19(9):e1010934. doi: 10.1371/journal.pgen.1010934 (PMC10564121; doi:10.1371/journal.pgen.1010934)
Supplement: Attachment — Submitted filename: Height_plus_LDL_Deviators_Revision_Two_Comments_Final.docx [file pgen.1010934.s005.docx]

**Response to Reviewer Comments (PGENETICS-D-23-00163)**

We would first and foremost like to thank the reviewers again – we are extremely pleased that they feel we have properly addressed the majority of their comments. For the final minor changes, we have adjusted the manuscript accordingly, and commented on the changes below. Changes in the manuscript have been highlighted in yellow.

*Reviewer #2: I thank the authors for the effort placed on making a thorough revision of their paper which address well the reviewer comments.

Yet, I consider my second point (i.e., possibility of sample swaps explaining the polygenic deviation) requires minor revision, plus one consideration about the scope of the findings:

1. The text in the discussion section of the manuscript (pg 13/21 lines 313-316) is incomplete and with a typo: "Fourth, a potential explanation for polygenic deviation is sample mix-up and [17]. However, we did not apply methods to determine this because 1) some methods rely on phenotypic mismatch with polygenic expectation, and 2) we have used samples that have not been flagged by UK Biobank as having sex-mismatches."*

Thank you for spotting this typo – we have corrected the manuscript. *2. In relation to the previous point, the question "whether the reported polygenic deviations consequence of sample swaps or not?", is not fully answered. While cross-sex sample swaps may have been identified, same-sex swaps will run undetected and the numbers (0.15% for height and 0.12% for LDL-C) seem consistent with those few who may have escaped the QC control. Rather, specify in the discussion that the deviants in the height analysis are NOT the same individuals of the LDL-C analysis; just as Idefix works by identifying those individuals that behave as deviants across multiple PRS's.*

We have further clarified that the individuals who we classified as deviating from their polygenic score are not the same, as well as not being cross-sex samples.  *3) The table provided for my raised point 3 with subject characteristics across LDL-C quintiles (in women with lower measued LDL-C) lacks the sample size (n) of deviators per quintile. I could predict, Q4 is where the largest amount of deviant is given that it holds the biggest contrast in age is observed, correct?. Strikingly, all deviants (independent of quintile) show rather consistently lower BMI, alcohol use and smoking. This is somewhere marginally stated by the authors along the manuscript. Yet, this also means a healthy lifestyle "overrides" the genetic predisposition... don't you want to emphasise more that message? In the conclusion, authors focus only on one perspective: "These individuals are more likely to carry rare genetic variation, or be at greater risk of co-morbidities, and should be considered in future discovery studies." You can also emphasise such positive perspective that has great potential to catch media attention. ;)*

Thank you for raising this point and agree that specific lifestyle characteristics may also contribute towards deviation from polygenic deviation. We have added the following to the discussion (page 12, lines 280-281):

*“Second, individuals who deviated were also enriched for other factors known to be associated with differences in phenotype, such as differences in BMI, smoking, and socio-economic position for LDL-C, consistent with the expectation that lifestyle factors play a role in deviation from polygenic prediction.”*

We apologize for not having presented the N in the table shown before but can report the number of females with lower LDL-C than expected in quintiles 1,2,3,4 and 5 of LDL-C polygenic score are 15, 1, 4, 2 and 19 respectively.
